# A New Methodology for the Accurate Measurement of Tibiofemoral Kinematics in Human Cadaveric Knees: An Evaluation of the Anterior–Posterior Laxity Pre- and Post-Cruciate Ligament Resection

**DOI:** 10.3390/life14070877

**Published:** 2024-07-14

**Authors:** Saskia A. Brendle, Sven Krueger, Joachim Grifka, Peter E. Müller, Thomas M. Grupp

**Affiliations:** 1 Research & Development, Aesculap AG, 78532 Tuttlingen, Germany; 2 Department of Orthopaedic and Trauma Surgery, Musculoskeletal University Center Munich (MUM), Campus Grosshadern, LMU Munich, 81377 Munich, Germany; 3 Department of Orthopaedics, Asklepios Klinikum, 93077 Bad Abbach, Germany

**Keywords:** knee, biomechanics, cadaveric study, anterior–posterior stability, cruciate ligaments

## Abstract

Anterior–posterior (AP) stability is an important measure of knee performance after total knee arthroplasty (TKA). To improve the stabilizing effect of implants designed to compensate for the loss of the cruciate ligaments, it is important to understand the tibiofemoral contact situation within the native ligamentous situation of the knee and how it changes after cruciate ligament resection. This in vitro study introduces a new approach to accurately measure the tibiofemoral kinematics in a six-degrees-of-freedom joint motion simulator by tracking landmark-based coordinate systems and their corresponding bone geometries. The tibiofemoral contact situation was investigated by projecting the medial and lateral flexion facet centers onto the tibial plateau under AP shear forces across various flexion angles in thirteen knees. Tests were conducted pre- and post-cruciate ligament resection. Post-cruciate ligament resection, the femoral condyles shifted closer to or even exceeded the posterior border of the tibial plateau, but only slightly closer to the anterior border. This study presents a new methodology for measuring the tibiofemoral kinematics that can be applied to multiple loading profiles. It provides a basis for further investigations, including passive or active muscle forces, to enhance the design of total knee protheses and improve surgical outcomes.

## 1. Introduction

The decision to sacrifice or preserve the posterior cruciate ligament (PCL) in total knee arthroplasty (TKA) remains debated and depends mainly on the surgeon’s preference. In cases where the PCL is insufficient or absent, posterior-stabilized (PS) or ultra-congruent (UC) implants can be used. PS implants substitute the PCL with a post-cam mechanism, whereas UC implants provide stability through conformity. While some studies have shown that neither UC nor PS implants provide anterior–posterior (AP) stability in all the positions of flexion [1,2], other studies have reported good clinical results for both PS and UC implants [3,4,5,6]. Nevertheless, instability is still one of the most common indications for revision after TKA [7,8,9]. Since AP stability is an important measure of functional knee performance, understanding the tibiofemoral contact situation within the native ligamentous situation and its changes after cruciate ligament resection is crucial to analyze where the implant needs to provide a stabilizing effect to compensate for the loss of both cruciate ligaments. Biomechanical in vitro studies provide high accuracy and control, offering insights into the AP stability of the knee joint unattainable in vivo due to ethical constraints [10,11,12,13,14,15,16,17,18,19]. However, many studies lack detailed investigation of the position of the medial and lateral femoral condyles on the proximal tibia during AP shear forces, particularly without cruciate ligaments. This gap is due to the limitations of current methodologies, which often fail to connect coordinate systems to the corresponding bone geometries [11,13,18]. In addition, many studies were performed using mechanical knee rigs [11,15,16], which use muscle forces to induce movement and therefore cannot actively control each degree of freedom separately to reproduce scenarios without muscles, such as passive laxity measurements or passive knee flexion as performed intraoperatively, or by applying manual force [13], which has limited reproducibility. In addition, the application of forces during testing is an important aspect to ensure comparability between measurements. As the relationship between coordinate systems and bone geometries is often unknown prior to testing, force application along reliable axes cannot be controlled.

Therefore, the primary objective of this study was to develop a new methodology and testing workflow for controlled force application and accurate tracking of landmark-based femoral and tibial coordinate systems and their corresponding bone geometries in human cadaveric knees during static and dynamic testing in a six-degrees-of-freedom joint motion simulator. The secondary objective was to show an application of this workflow by simulating clinical knee examinations as performed intraoperatively and evaluating the positions of the medial and lateral femoral condyles on the tibial plateau during anterior and posterior shear forces pre- and post-cruciate ligament resection. The hypothesis was that, in the native condition in deep flexion, the lateral femoral condyle approaches subluxation at the posterior border of the envelope of laxity, whereas the medial femoral condyle does not. Post-cruciate ligament resection, we anticipated the positions of the femoral condyles to move close to the anterior and posterior border of the tibial plateau.

## 2. Materials and Methods

### 2.1. Specimens

Thirteen fresh-frozen human cadaveric lower right extremities, preserved from the femoral head to the malleoli, were included in this study. The donors had a mean age of 66.62 ± 9.97 years and a mean body mass index (BMI) of 23.31 ± 8.02. The sample included three females and ten males. Medical records indicated no pre-existing knee disorders or surgical interventions. Ethical approval was obtained from the ethics committee of the Ludwig Maximilian University of Munich (No. 20-0856).

### 2.2. Coordinate Systems

Computed tomography (CT) scans (Siemens SOMATOM Perspective, Siemens Healthineers, Erlangen, Germany) were obtained for each specimen. The scans were segmented using Mimics 24.0 (Materialise, Leuven, Belgium), and the anatomic landmarks were identified with 3-matic medical 16.0 (Materialise, Leuven, Belgium) to define the femoral and tibial coordinate systems and further relevant axes. 

**Figure 1:**
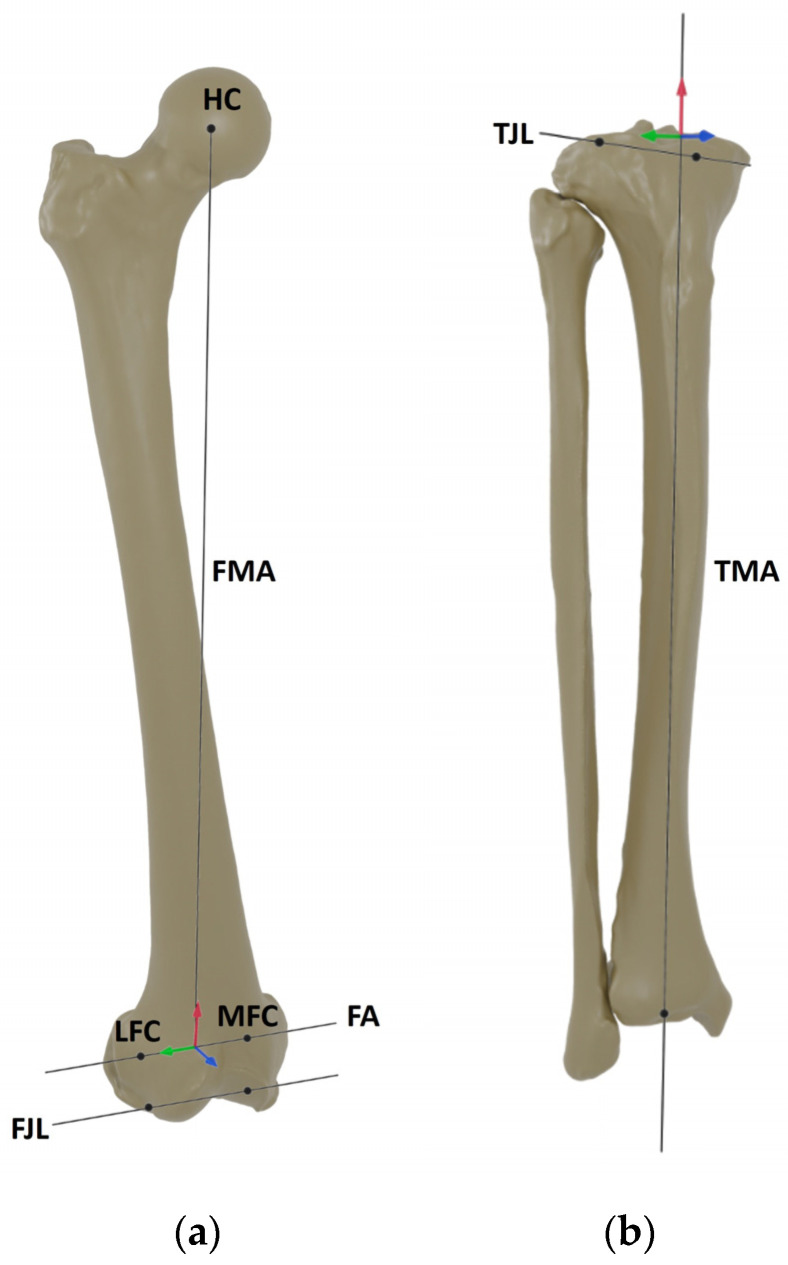
Anatomic landmarks and (**a**) femoral and (**b**) tibial coordinate systems. Anterior–posterior axes are marked in blue, medial–lateral axes are marked in green and proximal–distal (mechanical) axes are marked in red. FA = flexion axis, FJL = femoral joint line, FMA = femoral mechanical axis, HC = hip center, LFC = lateral flexion facet center, MFC = medial flexion facet center, TJL = tibial joint line, TMA = tibial mechanical axis.

For the femur, the flexion axis was defined by connecting the centers of two spheres fitted in the posterior condyles (medial flexion facet center (MFC) and lateral flexion facet center (LFC) [20,21], whereas the midpoint between the medial sulcus and the lateral prominence was specified as the origin of the femoral coordinate system. The center of the hip was determined by fitting a sphere in the femoral head. By connecting this point to the origin of the femoral coordinate system, the mechanical axis was identified [21]. The AP axis of the femoral coordinate system resulted from these two axes and pointed anteriorly. The femoral joint line was defined as a tangent through the most distal points of the medial and lateral condyles. For the tibia, a cone fit was applied in between the tibial tuberosity and the malleoli to identify the mechanical axis [22]. The proximal exit point of this axis was used as the origin of the tibial coordinate system. The AP axis of the tibial coordinate system was defined by a line connecting the attachment point of the PCL and the medial third of the tuberosity [23]. The medial–lateral axis of the tibial coordinate system resulted from the cross-product of the other two axes and pointed laterally. The tibial joint line was defined as a tangent through the most distal points on the medial and lateral tibial plateau [24]. Figure 1 illustrates the femoral and tibial coordinate systems.

### 2.3. Specimen Preparation

**Figure 2:**
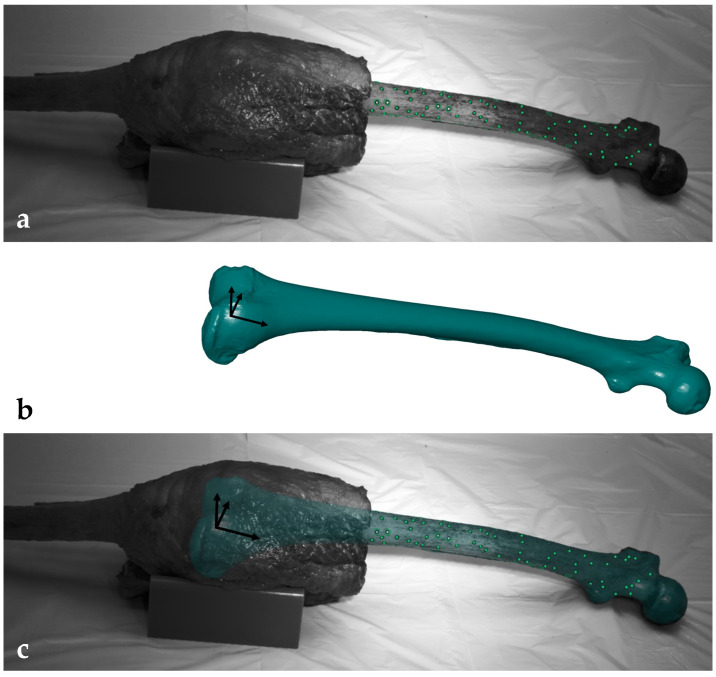
(**a**) Cadaveric femur with measuring points (green). (**b**) Segmented computed tomography (CT) scan (blue) with landmark-based femoral coordinate system. (**c**) Cadaveric femur with 3D-fitted segmented CT scan (blue).

Each specimen was thawed for 24 h at 7 °C before the experiment. The proximal and distal segments of the leg were skeletonized, preserving the knee joint capsule and surrounding soft tissue, including the ligaments, muscles and tendons, up to 100 mm superior and 50 mm inferior to the knee joint space. The GOM measuring points (1.5 mm, Carl Zeiss GOM Metrology GmbH, Braunschweig, Germany) were attached to the cleaned bone surfaces and the 3D point clouds of each bone were acquired using ARAMIS 12M (Carl Zeiss GOM Metrology GmbH, Braunschweig, Germany) by rotating the specimen stepwise around its mechanical axis. The 3D fittings of the femur and tibia were performed by aligning the segmented CT scans containing the landmark-based coordinate systems with the previously acquired 3D point clouds of each bone (Figure 2). To ensure accurate alignment, visual and quantitative inspections of the 3D fittings were conducted from different perspectives. The quantitative inspections were performed by palpating each bone at a minimum of six points with a touch probe (1 mm, PM 3, Carl Zeiss GOM Metrology GmbH, Braunschweig, Germany) and measuring the distance to the matched segmented CT scan. The mean deviations should not exceed ± 1 mm. After successful fitting, the 3D fitting information was saved and the specimens were transected 250 mm proximally and 150 mm distally to the knee joint space. The intramedullary canals were cleaned and closed with Play-Doh before potting into fast-cast resin (Gößl und Pfaff, Brautlach, Germany). 

For accurate measurement of the kinematics, it was necessary to place the femur in the joint motion simulator in such a way that the femoral coordinate system was aligned with the upper coordinate system of the joint motion simulator. For this purpose, the upper coordinate system was virtually assigned to the custom-made aluminum femur pot, as its fixed position in the joint motion simulator is known. This was achieved by performing a 3D fitting of the femur pot by aligning its computer-aided design (CAD) file containing the upper coordinate system of the joint motion simulator to the femur pot. To embed the femur, the specimen and the femur pot were placed in an embedding fixture. A 2D point cloud of the femur pot and the residual femur was acquired and the previously created 3D-fitting files of femur pot and femur were loaded again. Due to the unique point patterns, it was possible to realign the 3D-fitting files to the new 2D point cloud. In this way, the accurate 3D information of the complete femur and the according coordinate system was available, even after the femur was cut. The femur was adjusted while tracking the measuring points such that the femoral coordinate system was within ±0.5 mm and ±1° relative to the upper coordinate system of the joint motion simulator. After successful alignment, the proximal femur was embedded in the femur pot and attached to the custom-made aluminum fixture mounted on the upper actuator of the joint motion simulator. To embed the tibia, the upper actuator was held in position and an axial compression force of 100 N was applied to the specimen, with all the other forces and moments maintained at 0 N/Nm. In order to embed the tibia at 0° flexion, the mechanical axis of the tibia was aligned with the mechanical axis of the femur in the sagittal plane while tracking their coordinate systems using measurement points on the tibia and the tibia pot in combination with the previously created 3D-fitting files. The tibia and fibula were then potted together in a custom-made aluminum tibia pot that was fixed on the lower actuator of the joint motion simulator.

### 2.4. Experimental Testing

**Figure 3:**
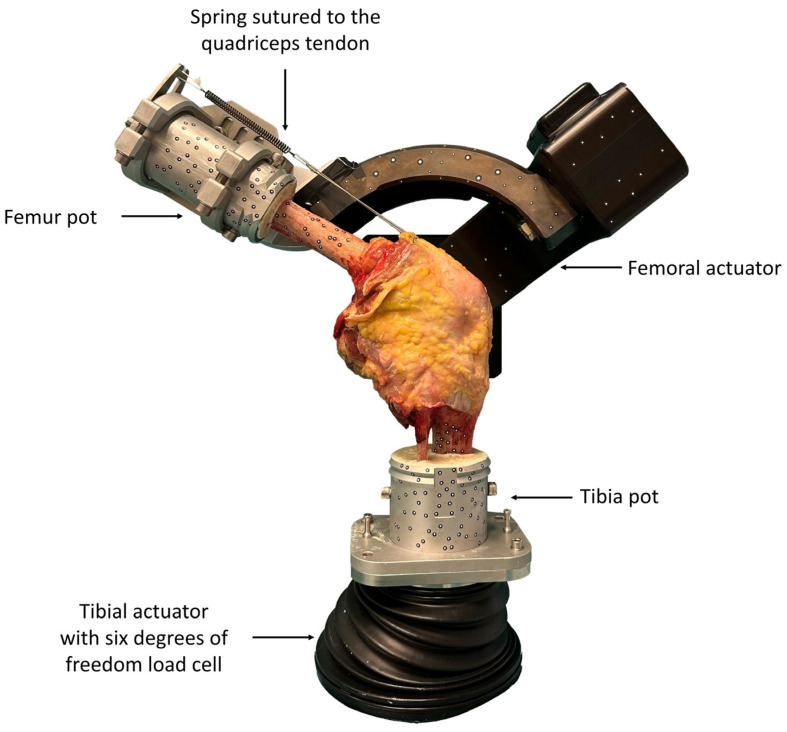
Experimental setup with the knee specimen mounted on the six-degrees-of-freedom joint motion simulator at 60° flexion.

Testing was conducted using a six-degrees-of-freedom joint motion simulator (VIVO, Advanced Mechanical Technologies Inc., Watertown, MA, USA). The six degrees of freedom were implemented by two actuators: the upper (femoral) actuator and the lower (tibial) actuator. The simulator’s upper actuator performed flexion–extension and varus–valgus rotations, while the lower actuator managed medial–lateral, anterior–posterior, proximal–distal translations, and internal–external rotation. Each degree of freedom could be controlled in force or displacement mode independently. The forces and motions are expressed in accordance with the conventions of Grood and Suntay [25]. Figure 3 provides an overview of the experimental setup.

**Figure 4:**
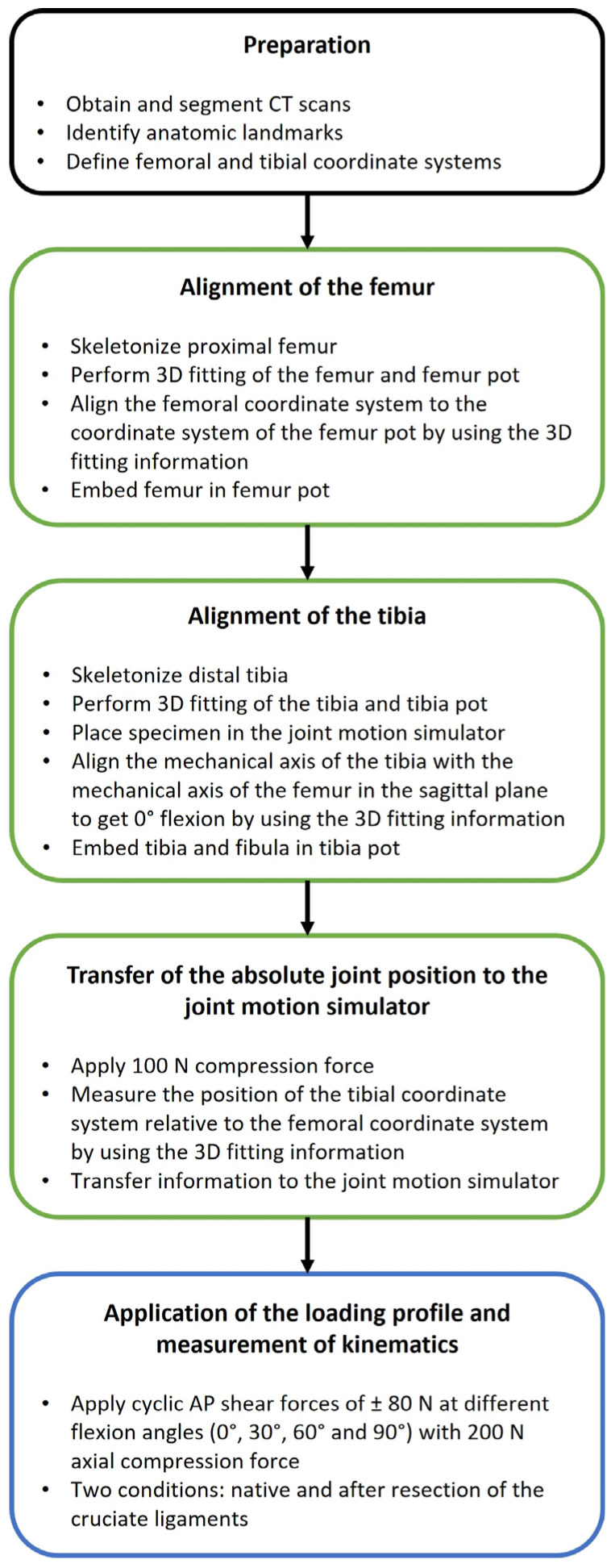
Flowchart illustrating the key steps in the entire test process. The preparation is marked in black. The workflow to ensure accurate measurement of the tibiofemoral kinematics is marked in green. The application of the loading profile and measurement of the kinematics is marked in blue.

To transfer the absolute joint position of the specimen to the joint motion simulator, a reference position was recorded under an axial compression force of 100 N at 0° flexion, with all the other forces and moments maintained at 0 N/Nm. At the same time, the previously generated 3D-fitting information of the segmented CT scans were again projected onto the residual bones using the remaining measuring point patterns. Based on this information, the relative position of the femoral and tibial coordinate systems was recorded and transferred to the joint motion simulator. Afterwards, the specimens were subjected to laxity testing. In order to characterize the passive AP laxity of each knee in the native condition, cyclic AP shear forces of ±80 N were applied as a ramp profile for 4 cycles at different flexion angles (0°, 30°, 60° and 90°) at a frequency of 0.04 Hz while maintaining an axial compression force of 200 N and all the other forces and moments at 0 N/Nm. The imposed loads were chosen to reflect those applied during clinical examination of joint laxity and were within the range of forces used in comparable studies [11,15,16]. After the native measurements, the cruciate ligaments were transected and the measurements were repeated. For both conditions, the knee capsule was opened using a medial parapatellar approach and closed with surgical sutures (Number 1 Vicryl, B. Braun, Melsungen, Germany) to ensure that only the effects of the cruciate ligament resection were investigated and to exclude the effects of opening the capsule. During testing, the relative position of the femoral and tibial coordinate systems and the resulting loads were sampled at 100 Hz by the joint motion simulator. To simulate the passive tension of the patella tendon in flexion, as present during the intraoperative clinical examination, a spring with an increasing force of up to 50 N at 90° flexion was sutured to the quadriceps tendon with a line of action parallel to the anatomical axis of the femur. Furthermore, the specimens were kept moist by spraying the tissue with sodium chloride solution in order to mitigate the effects of tissue drying during the experimental testing. Figure 4 outlines the entire test process.

### 2.5. Data Analysis

**Figure 5:**
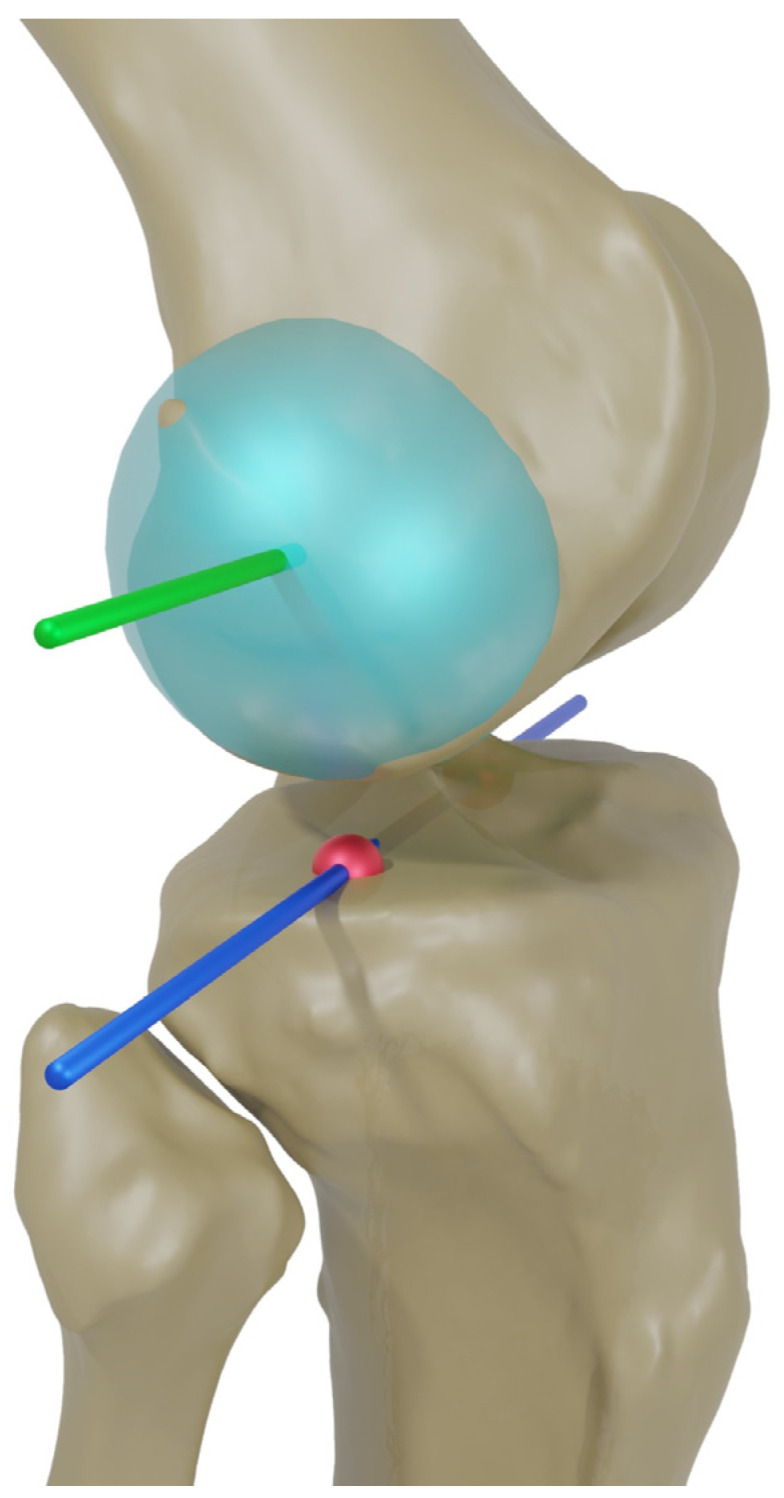
Schematic illustration of the fitted spheres (light blue) in the posterior condyles and the projection (blue) of the flexion axis (green) as well as the medial and lateral flexion facet centers (MFC and LFC, red) ono the tibial plane.

Data were analyzed using MATLAB (Version R2023a, MathWorks Inc., Natick, MA, USA). The first and last cycles of each measurement were removed from the data analysis to prevent potential data loss due to the start and stop behavior. Furthermore, the kinematic output of the second and third cycles showed excellent agreement (Table 1), and therefore, only data from a single cycle of each experiment were investigated. To evaluate the control accuracy, the root mean square error (RMSE) of all six controlled degrees of freedom was calculated by taking the square root of the mean of the squared differences between the target and the actual values for all the measurements. The mean deviation between the bone and the segmented CT scan was calculated, ensuring accuracy within ±1 mm. Furthermore, the MFC and LFC of each knee were projected onto the articular surface of the proximal tibia at different flexion angles (0°, 30°, 60° and 90°) at the first time reaching the maximum anterior and posterior force, respectively, reflecting the corresponding positions of the medial and lateral condyles on the tibial plateau (Figure 5). Subsequently, the positions were normalized to the AP width of the articular surface of the respective tibia, which was defined as the distance between the most anterior and the most posterior point of the medial compartment of the tibia.

Three specimens were removed from the data analysis due to exceeding the motion limits of the joint motion simulator or joint luxation. The statistical analyses were performed using Minitab (Version 21.2, Minitab GmbH, Munich, Germany). The significance of the differences between the positions in the native condition and the condition after resection of the cruciate ligaments was determined for each condyle at the flexion angles of 0°, 30°, 60° and 90° using Wilcoxon signed-rank tests with the significance level set at *p* ≤ 0.05. The results are displayed with boxplots on a normalized tibia.

## 3. Results

### 3.1. Accuracy of 3D Fittings

**Figure 6:**
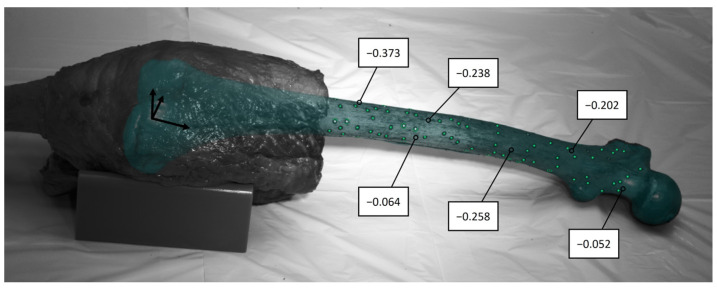
Cadaveric femur with measuring points (green), 3D-fitted segmented CT scan (blue) and deviations between the real bone and the 3D-fitted segmented CT scan at specific points. All the deviations are displayed in mm.

Quantitative inspections of the 3D fittings using a touch probe revealed a mean deviation of |0.27 ± 0.21| mm between the real bone and the 3D-fitted segmented CT scan (Figure 6). Most deviations were negative, indicating that the segmented CT scan slightly protruded beyond the real bone at these locations.

### 3.2. Control Accuracy

**Figure 7:**
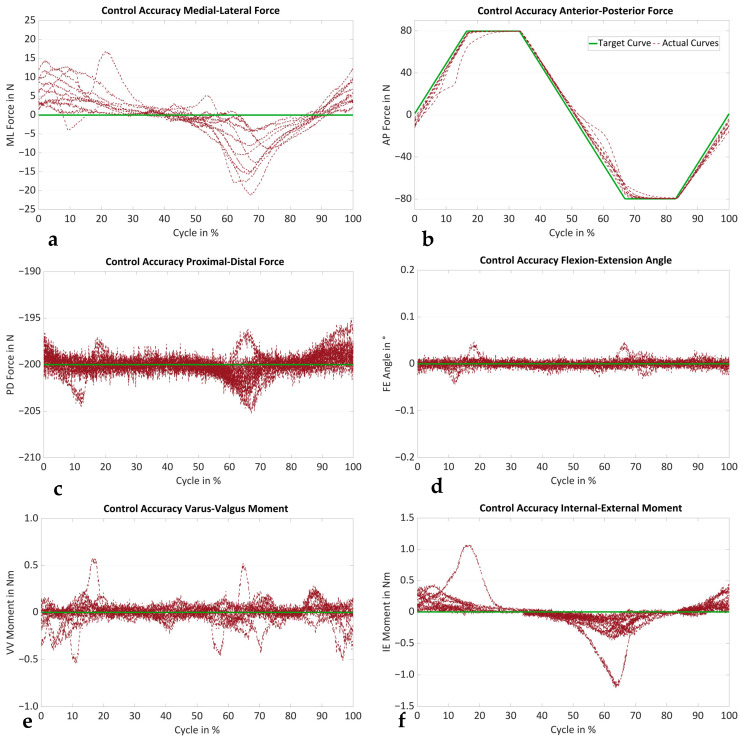
Target curve (green) and actual curves (red) of the (**a**) medial–lateral (ML) force, (**b**) anterior–posterior (AP) force, (**c**) proximal–distal (PD) force, (**d**) flexion–extension (FE) angle, (**e**) varus–valgus (VV) moment and (**f**) internal–external (IE) moment of all the specimens (*n* = 10) in the native condition at 0° flexion, showing the control accuracy.

The RMSE of the control error of all six degrees of freedom for all the measurements is summarized in Table 2. The RMSEs of the flexion–extension (FE) angle, varus–valgus (VV) and internal–external (IE) moments, and the proximal–distal (PD) force were small. The largest errors were observed in the medial–lateral (ML) and AP directions. Figure 7 shows one cycle of the target and the actual curves of all six controlled degrees of freedom for all the specimens in the native condition at 0° flexion. Again, the good control accuracy of the FE angle, as well as the VV and IE moments, and the PD force can be seen. The largest deviations from the target curve were observed for the AP and ML. However, the target AP forces of ±80 N were achieved for all the specimens.

### 3.3. Influence of the Cruciate Ligaments on the Anterior–Posterior Laxity

**Figure 8:**
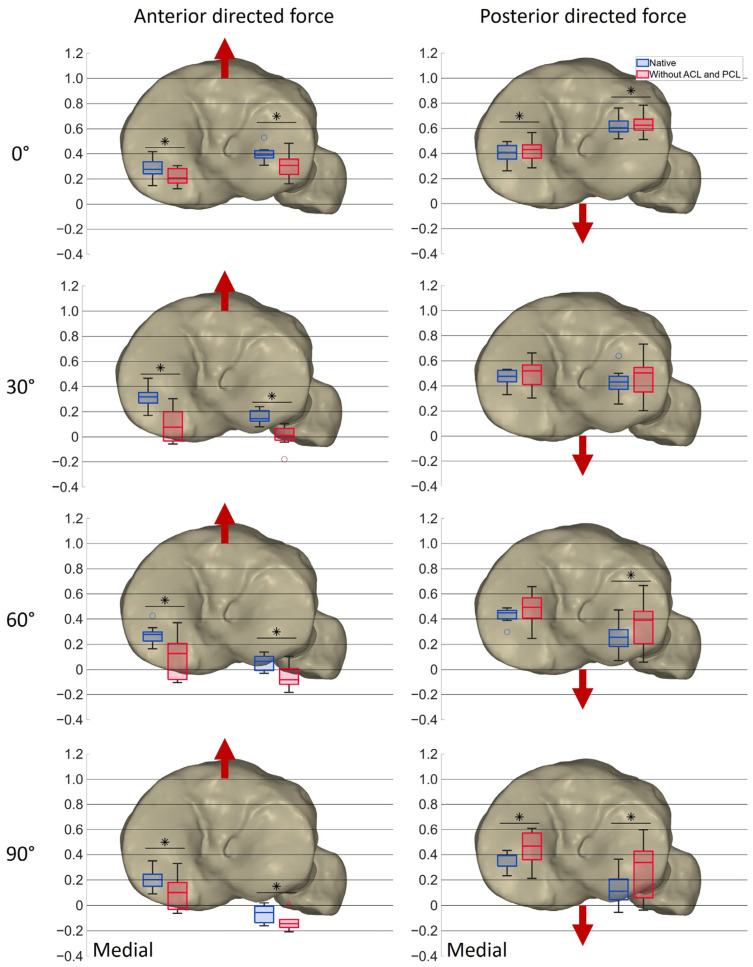
Boxplots showing the median (*n* = 10), first and third quartile, range and outliers of the AP positions of the projected MFC and LFC at different flexion angles at the maximum anterior and posterior force, respectively, in the native condition (blue) and after resection of the cruciate ligaments (red) on a normalized tibia. Red arrows indicate the direction of the shear force applied on the tibia. Significant differences are marked with an asterisk (*p* ≤ 0.05).

Figure 8 shows the AP positions of the projected MFC and LFC at various flexion angles under the maximum anterior and posterior shear force acting on the tibia, pre- and post-cruciate ligament resection. The boxplots based on a normalized tibia illustrate these positions with the medians, quartiles, ranges and outliers. Significant differences (*p* ≤ 0.05) are marked with an asterisk. 

In the native condition, the median position of the medial condyle under anterior force was located in the posterior third of the tibia at 0° flexion (0.28), shifted slightly anteriorly at 30° flexion (0.31), and reached its most posterior position at 90° flexion (0.20). Under posterior force, the medial condyle’s median position was located 0.41 from the posterior border of the tibial plateau at 0° of flexion, moved anteriorly at 30° flexion (0.48), and returned to near its initial position at higher flexion angles. The median position of the lateral condyle in the native condition was more anterior than the medial condyle for both anterior (0.39) and posterior (0.60) force at 0° flexion and shifted posteriorly throughout the arc of flexion, already exceeding the posterior border of the tibial plateau at 90° flexion with the maximum anterior force (−0.06). The lateral condyle thus showed a noticeably greater change in position during flexion than the medial condyle, which can be interpreted as a medial pivoting kinematic. 

After cruciate ligament resection, the median position of the medial condyle shifted significantly more posteriorly under anterior force at all the flexion angles: 0° (0.21, *p* = 0.006), 30° (0.08, *p* = 0.006), 60° (0.13, *p* = 0.006), and 90° (0.10, *p* = 0.006). The median position of the lateral condyle also shifted posteriorly and was significantly more posterior than in the native condition at 0° (0.31, *p* = 0.006), 30° (0.01, *p* = 0.006), 60° (−0.08, *p* = 0.006) and 90° (−0.14, *p* = 0.008) of flexion. Under posterior force, the medial condyle’s median position was significantly more anterior at 0° (0.43, *p* = 0.006) and 90° (0.47, *p* = 0.032) of flexion, while the lateral condyle showed significant anterior shifts at 0° (0.63, *p* = 0.008), 60° (0.39, *p* = 0.008) and 90° (0.34, *p* = 0.006). 

The results suggest that resection of the cruciate ligaments significantly alters the anterior–posterior stability of the knee, with both femoral condyles showing increased posterior displacement under anterior force and varied behavior under posterior force. The increased length of the boxplots after resection of the cruciate ligaments indicates greater variability, especially at higher flexion angles.

## 4. Discussion

In this study, a new workflow was developed for accurately tracking landmark-based femoral and tibial coordinate systems and their corresponding bone geometries in human cadaveric knees during static and dynamic testing using a six-degrees-of-freedom joint motion simulator. 

A reliable method to define coordinate systems is crucial in kinematic assessments, as different coordinate systems can lead to significant variations in the results and interpretations of normal movement [26,27]. For this reason, landmark-based coordinate systems are recommended for measuring tibiofemoral kinematics [26]. However, accurate and reliable placement of landmark-based coordinate systems in cadaveric knees is challenging due to soft tissue covering the landmarks. Moreover, if the landmarks are palpated using a touch probe, from which a coordinate system is finally formed, the exact relationship between the bone and the coordinate system is not known. In the workflow developed in the scope of this study, the landmarks are selected in 3D models of the bones using geometrical primitives that have been shown to have a good inter-observer reliability [22,28], allowing for precise and reproduceable placement. In addition, the accuracy of the subsequent 3D fitting can be analyzed by measuring the deviation between the bone and the fitted segmented CT scan using a touch probe. Small deviations may result from the quality of the CT scan, segmentation errors or the possibility of scraping bone during preparation. The high accuracy of the 3D fitting in this study and the knowledge of the relative positions of the femoral and tibial bone geometries enable multiple analyses, such as projecting the flexion axis and flexion facet centers (FFCs) onto the tibial plane. This method approximates the tibiofemoral contact pattern [16,29,30] and is valid to describe the kinematic behavior of the knee [31], especially for single radius implant designs [32]. Fitting of the spheres into the posterior condyles in the present study revealed that the specimens mainly had a constant radius of curvature.

Another important aspect during static and dynamic testing is the application of forces. If the exact relationship between the bone and the coordinate system is not known before testing, it cannot be ensured that the forces during testing are applied along the same axes when comparing different specimens. In the workflow developed in this study, not only the location of the tibial coordinate system with respect to the femoral coordinate system but also their location relative to the bones is known. This is a major advantage of the method, as it enables the controlled application of forces and moments through the origin of the tibial coordinate system with the line of action parallel to the Grood and Suntay axes [25,33]. This ensures consistent and reliable force application across different specimens. Furthermore, the six-degrees-of-freedom joint motion simulator allows for independent control of each degree of freedom, making it possible to reproduce scenarios without muscle forces, such as passive laxity measurements or passive knee flexion as performed intraoperatively. This capability is relevant for navigation and robotics research. In addition, it is possible to apply passive knee flexion with a controlled preload or even complex loading scenarios like level walking based on the CAMS knee dataset [34]. Moreover, the simulator can be modified by adding virtual ligament models and actively controlled quadriceps and hamstrings forces [17,33,35]. Previous studies have shown that adding the muscle load significantly affects the resulting kinematics [17,36]. Depending on the research question, it may therefore be more appropriate to perform active instead of passive movements. The present study focused mainly on characterizing the tibiofemoral contact situation within the ligamentous situation. For this reason, passive movements were chosen, since forces across the joint reduce the contribution of the soft tissue and increase the contribution of the articular surfaces [37]. However, the results may be different when loading the knee with hamstrings and quadriceps force [36]. Nevertheless, this workflow provides a basis for reliable force application and tracking of the resulting kinematics, regardless of which loading profile is applied. In addition, the six-degrees-of-freedom joint motion simulator demonstrated good control accuracy for the loading profile used in this study, as well as kinematic reproducibility, as evidenced by the excellent agreement of the kinematic output between two cycles. However, the control parameters may need to be adjusted for more complex loading profiles.

In order to accomplish the secondary objective of the present study, the new workflow was used to evaluate the positions of the medial and lateral femoral condyles on the tibial plateau during anterior and posterior shear forces in the native knee and after resection of both cruciate ligaments. In the native condition, the lateral femoral condyles moved posteriorly on the tibial plateau with increasing flexion under both anterior- and posterior-directed forces. The medial femoral condyles moved slightly anteriorly until 30° of flexion under posterior-directed force and then continued moving posteriorly until 90° of flexion. The results support the hypothesis that with anterior-directed force in deep flexion, the lateral femoral condyle approaches the posterior border of the tibial plateau, while the medial femoral condyle remains more anterior. Moreover, the results are in accordance with a previous study [16] and reflect the medial pivoting characteristics that can be observed during the neutral path of motion [16,29,30]. 

The second hypothesis regarding the position of the femoral condyles after resection of the cruciate ligaments could not be confirmed. The positions of the femoral condyles shifted closer to or even exceeded the posterior border of the tibial plateau, but only slightly closer to the anterior border. This implies that the implant primarily has to provide posterior stabilization to compensate for the loss of both cruciate ligaments. However, the AP position of the femoral condyles on the tibial plateau was shown to vary depending on the axial force and tibial slope [12,15]. Previous studies reported a more posterior position and less anterior translation with an increasing slope [12]. The anatomical tibial slope in the specimens, with a mean medial slope of 9.14 ± 2.81° and higher lateral slopes in most cases, might explain why the femoral condyles are closer to the posterior tibial border. For this reason, the positions of the femoral condyles on the tibial plateau might be different with less slope, as would probably be the case after implantation of tibial total knee components. Depending on the prothesis and alignment technique, tibial components are usually implanted with less tibial slope than in the native situation, with recommendations ranging from 0° to 10° [38,39]. A study by Giffin et al. [12] showed that, even though more posterior translation of the femur was observed with increased slope, the overall AP translation remained the same. This implies that after resection of the cruciate ligaments, stabilization of the joint by the implant is necessary, even with less slope. Nevertheless, the stabilizing effect that the implant needs to provide may vary depending on the slope. Furthermore, various loading conditions may also lead to different results [36,40]. While this study mainly investigated the passive AP laxity within the ligamentous situation, the AP translation may vary during active movements including quadriceps and hamstrings force. For this reason, further research is needed to identify the stabilizing effects that implants must provide to compensate for the loss of both cruciate ligaments during various activities. Future studies should also consider the influence of different tibial slopes and physiological muscle loading to better understand the differences in knee kinematics pre- and post-implantation.

Several limitations should be taken into account when interpreting the results of the present study. First, this study investigated a limited number of human cadaveric specimens, which may not fully represent the variability found in living patients. Additionally, physiological muscle loading was not applied, and the patellar mechanism was only present to a limited extent by simulating the passive tension of the patella tendon in flexion, as present during intraoperative clinical examination. Therefore, the results may not be directly transferable to in vivo conditions and may only be representative of passive laxity tests as performed intraoperatively. However, it is essential to perform biomechanical in vitro studies to better understand the causes of a certain behavior, unattainable in vivo due to ethical constraints. Furthermore, AP laxity was not investigated beyond 90° of flexion due to the design constraints of the joint motion simulator; therefore, no conclusions can be drawn about laxity behavior at higher flexion angles. However, a large proportion of activities of daily living are covered with flexion angles of up to 90° [34]. Moreover, the order of the two different test conditions could not be changed. Time-dependent effects can therefore not be eliminated. The limited number of tests and the resulting short test duration should minimize these effects [41].

## 5. Conclusions

This study introduced a new method to accurately measure the tibiofemoral kinematics using a six-degrees-of-freedom joint motion simulator and landmark-based coordinate systems. The method was employed to analyze the positions of the medial and lateral femoral condyles on the tibial plateau under AP shear forces, pre- and post-cruciate ligament resection. The findings indicated that post-resection, the femoral condyles’ positions shifted close to or even exceeded the posterior border of the tibial plateau, but only slightly closer to the anterior border. In addition, the high accuracy of the 3D fittings, kinematic reproducibility and control accuracy were demonstrated. These findings highlight the method’s potential for further investigations to enhance the design of total knee protheses and improve surgical outcomes.

## Figures and Tables

**Table 1:** Root mean square error (RMSE) of the kinematic output between the second and third cycles of all the measurements for medial–lateral (ML), anterior–posterior (AP), proximal–distal (PD), flexion–extension (FE), varus–valgus (VV) and internal–external (IE) directions.

ML Translation [mm]	AP Translation [mm]	PD Translation [mm]	FE Rotation [°]	VV Rotation [°]	IE Rotation [°]
0.07	0.11	0.04	0.01	0.03	0.16

**Table 2:** RMSE of the control error for all the measurements for the medial–lateral (ML), anterior–posterior (AP), proximal–distal (PD), flexion–extension (FE), varus–valgus (VV) and internal–external (IE) directions.

ML Force Error [N]	AP Force Error [N]	PD Force Error [N]	FE Angle Error [°]	VV Moment Error [Nm]	IE Moment Error [Nm]
10.77	10.99	2.46	0.01	0.18	0.24

## Data Availability

The data presented in this study are available on request from the corresponding author. The data are not publicly available due to ethical and privacy considerations associated with human cadaveric donor material.
